# Melanoma With an Unknown Primary in an Asymptomatic Elderly Male With Unilateral Lymphadenopathy

**DOI:** 10.7759/cureus.15140

**Published:** 2021-05-20

**Authors:** Michael B Phan, Jonathan Phan, Chris Nguyen, Jing He, Quan D Nguyen

**Affiliations:** 1 Department of Dermatology, University of Texas Medical Branch, Galveston, USA; 2 Department of Anesthesiology, University of Texas Medical Branch, Galveston, USA; 3 Department of Pathology, University of Texas Medical Branch, Galveston, USA; 4 Department of Radiology, University of Texas Medical Branch, Galveston, USA

**Keywords:** melanoma with an unknown primary, melanoma

## Abstract

Melanoma with an unknown primary (MUP) is an uncommon metastatic melanoma without an obvious primary site. MUP has a higher prevalence in men in their fifth decade of life. The pathogenesis of MUP is still unknown but several hypotheses have been proposed including the predominant regression theory, occult cutaneous, or visceral location, or by the presence of ectopic melanocytes. Proper physical examination, imaging, and histopathological review are needed to diagnose MUP. Patients with MUP must be aggressively treated and monitored for recurrence. We present a case of MUP occurring in an asymptomatic 61-year-old male with axillary lymphadenopathy. We hope to raise awareness that melanoma of unknown primary can present in lymph nodes without external structural changes.

## Introduction

Melanoma with an unknown primary (MUP) is a metastatic melanoma without an obvious primary site. They are uncommon and the incidence varies between 3-10% [1,2]. MUP has a higher prevalence in men in their fifth decade of life [3]. The American Joint Committee on Cancer classified MUP as stage III if presented with lymph node or subcutaneous involvement or stage IV with visceral involvement [4]. MUP and melanoma with known primary (MKP) present with a similar course of disease and require aggressive surgical involvement and adjunct therapies [5]. The pathogenesis of MUP is unknown, but several hypotheses have been proposed including the predominant regression theory, occult cutaneous or visceral location theory, or by the presence of ectopic melanocytes theory [3,6,7]. We present a case of MUP occurring in an asymptomatic 61-year-old male with axillary lymphadenopathy. We hope to raise awareness that melanoma of unknown primary can present in lymph nodes without external structural changes.

## Case presentation

**Figure 1:**
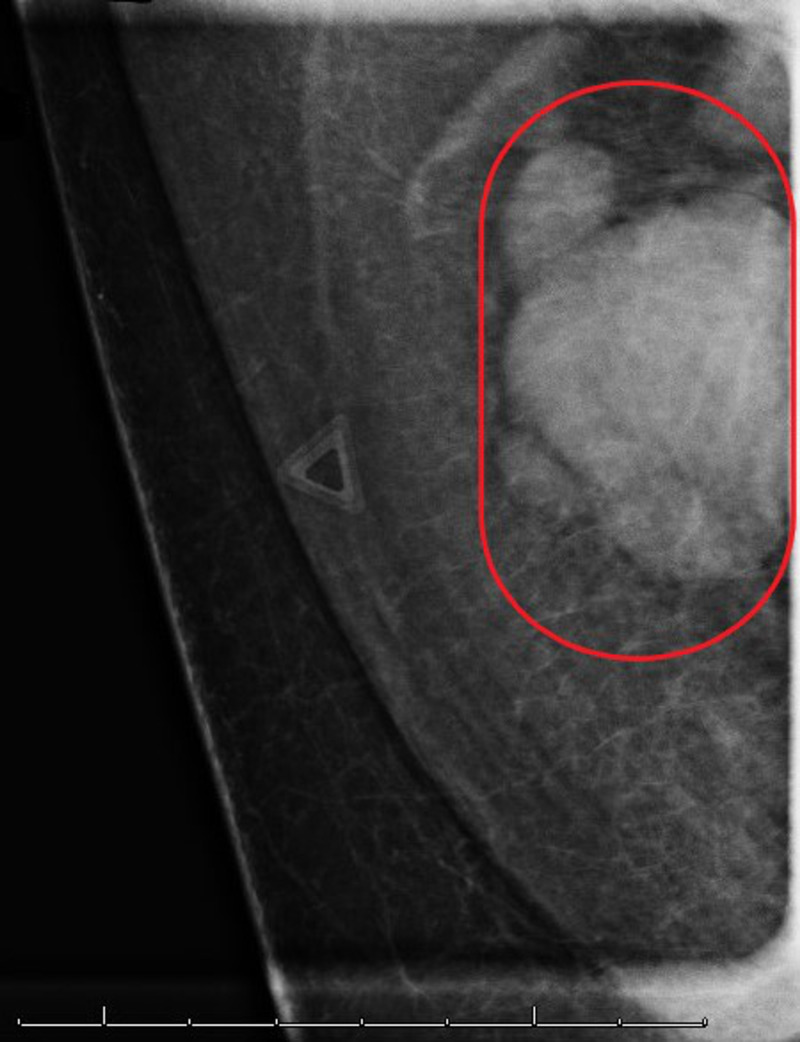
Initial Right Axilla Mammogram Image shows abnormal lymph nodes (red oval) with cortical thickening and loss of fatty hilum.

A 61-year-old Caucasian male presented to our clinic with a two-week history of a palpable and painless lump without drainage or erythema in the right axilla. He denied any symptoms of fever, chills, weight change, recent illness, or recent rash. He had not previously noted any changes in skin color and tone, or any other fluctuant masses on self-examination. A bilateral diagnostic mammogram found no suspicious masses, calcifications, or masses in the left breast, left axilla, or right breast. However, there was an irregular lymph node over the right axilla (Figure 1).

**Figure 2:**
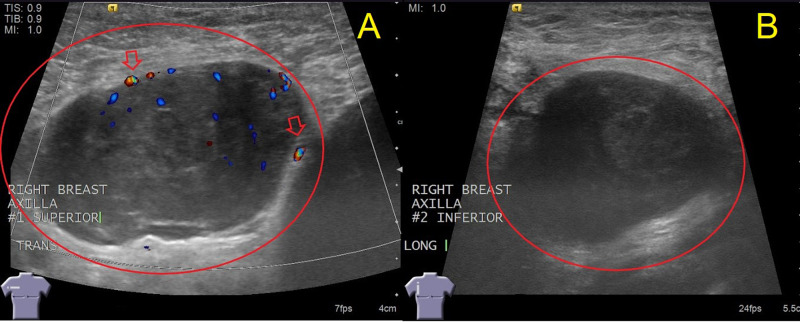
Right Axilla Ultrasound Images show a 37 mm x 24 mm x 40 mm irregularly shaped axillary lymph node (A) with an associated increased vascularity (arrows) and a 35 mm x 31 mm x 39 mm irregularly shaped adjacent lymph node (B) immediately inferior to lymph node A. Irregular lymph nodes are indicated with red circles.

**Figure 3:**
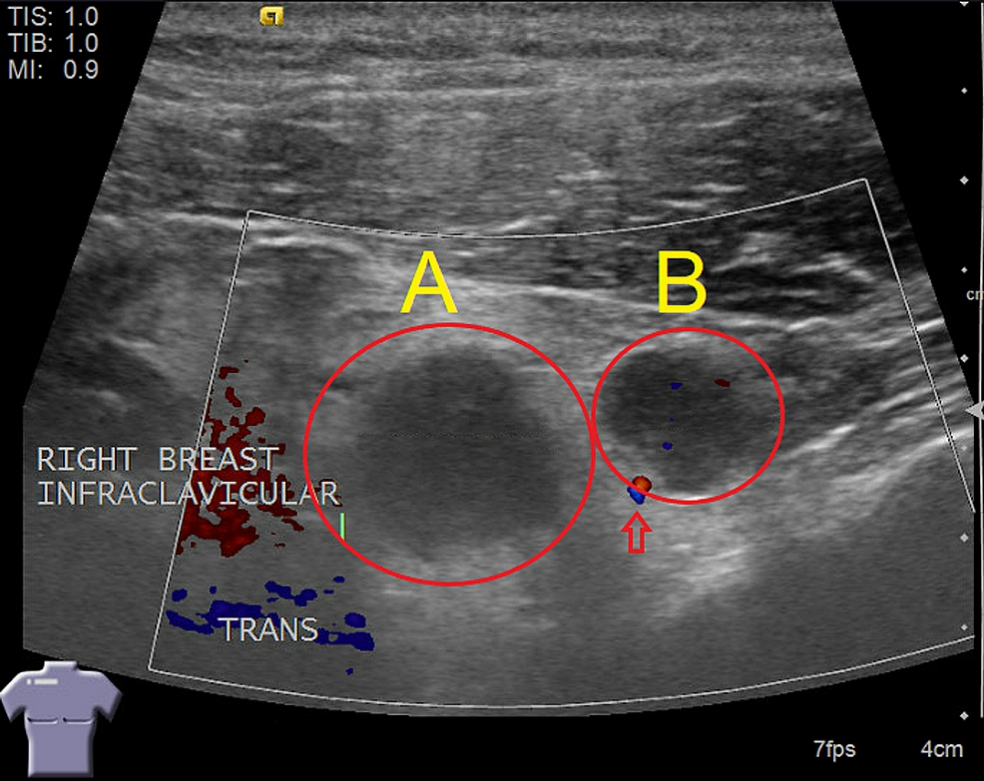
Right Infraclavicular Ultrasound Image shows two adjacent abnormal appearing infraclavicular lymph nodes (red circles) which measure 10 mm x 9 mm x 12 mm (A) and 8 mm x 10 mm x 12 mm (B) with mild associated increased vascularity (arrow).

**Figure 4:**
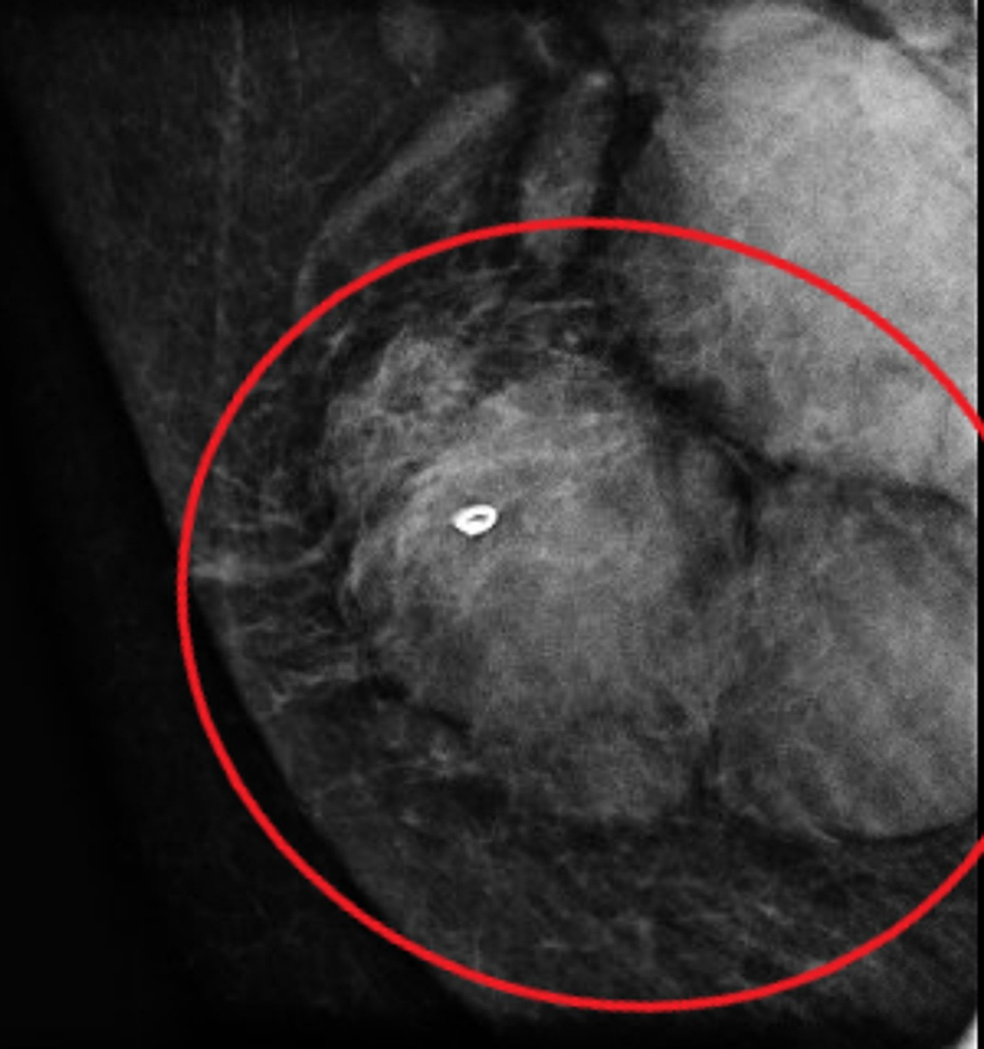
Mammogram Core Needle Biopsy Image shows two adjacent irregular masses (red circle).

Given the radiologic findings on the mammogram, a bilateral ultrasound of the breast was then obtained. The ultrasound showed two adjacent irregular lymph nodes in the right axilla (Figure 2). Ultrasound imaging also detected two abnormal infraclavicular lymph nodes (Figure 3). Because of these abnormalities, an ultrasound-guided core biopsy of the right axilla was performed to rule out lymphoma (Figure 4).

**Figure 5:**
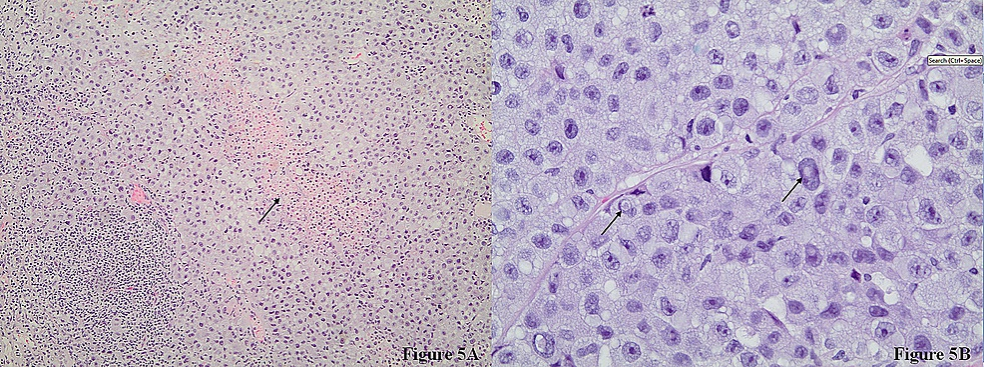
Histological Findings of Biopsy of Right Axillary Lymph Node (A) An ultrasound-guided core biopsy of the right axillary mass shows nests and sheets of poorly cohesive malignant tumor cells infiltrating the lymph node. Focal area of tumor necrosis is seen (arrow) (magnification x100). (B) Tumor cells demonstrate nuclear pleomorphism with large eosinophilic nucleoli, nuclear pseudoinclusions (arrow), mitotic figures, and abundant eosinophilic finely granular cytoplasm (magnification x400).

**Figure 6:**
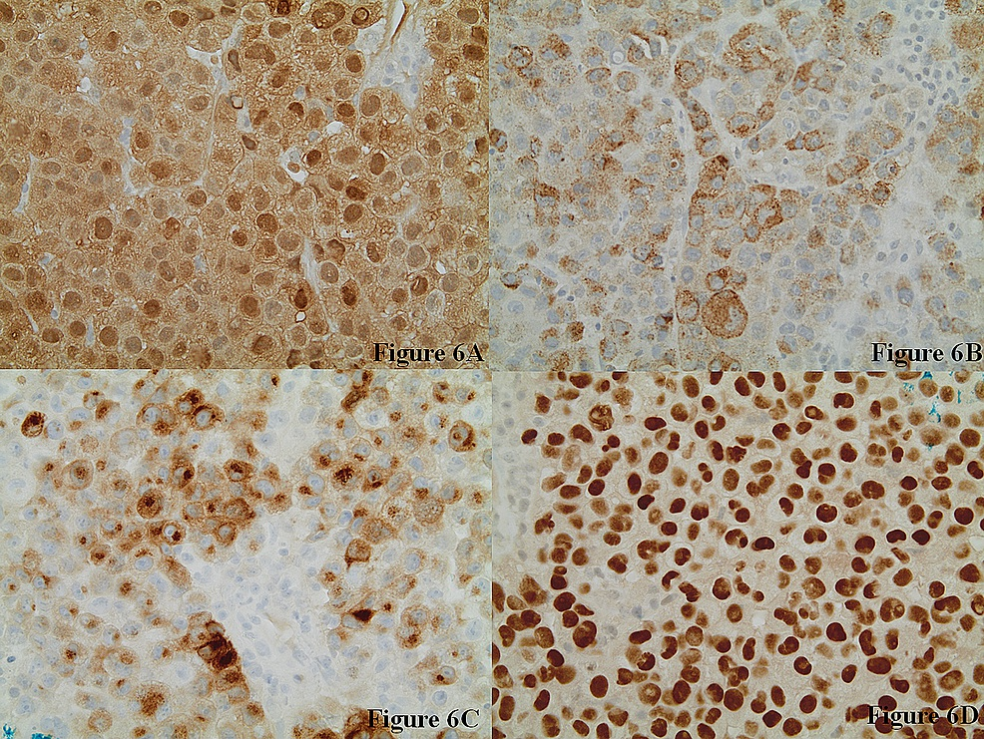
Immunohistochemical Stains at 400x Magnification Images show that the tumor cells are positive for S-100 (6A), HMB-45 (6B), Melan-A (6C), and Sox-10 (6D).

Histopathology revealed nests and sheets of poorly cohesive malignant tumor cells infiltrating the lymph node with nuclear pleomorphism and mitotic figures (Figure 5). Immunostaining was performed on the specimen and yielded a positive S100, positive HMB-45, positive Sox10, positive Melan-A, negative AE1/AE3, and negative P63 (Figure 6). Molecular studies were performed, which demonstrated a BRAF v600 positive mutation. He was referred to dermatology for a skin examination and a primary lesion was not found. These findings support the diagnosis of malignant metastatic melanoma with an unknown primary.

He was referred to surgical oncology for evaluation and management, as well as medical oncology for neoadjuvant therapy. He was started on dabrafenib 150 mg BID and trametinib 2 mg daily while awaiting surgery. On follow-up appointments, he tolerated the therapy and had near-complete resolution of his axillary lymphadenopathy. A right axillary lymphadenectomy was successfully performed. Surgical pathology of 28 lymph nodes showed fibrosis and histiocytic reactions consistent with treatment-related changes. He continued follow-up appointments with medical oncology and is currently in remission.

## Discussion

Melanomas with an unknown primary are uncommon. Studies have found that 3% of all melanomas are diagnosed with an unknown primary and 5-10% of patients with metastatic melanoma have a primary melanoma without a primary site [1,2]. In order to classify patients with MUP, Dasgupta et al. created a criteria to exclude patients with: 1) orbital exoneration or enucleation; 2) history of removal or cauterization of any skin lesions; 3) metastatic melanoma presenting with a scar in a draining lymph node from previous local treatment; 4) lack of a thorough physical examination [8,9].

The pathogenesis for MUP remains unclear. MUP occurs more often in men in their 40s-50s [3]. The predominant hypothesis involves the regression of melanoma from a known primary site [3]. Other theories have been suggested including the presence of melanomas in occult cutaneous or visceral location or by the presence of ectopic melanocytes from preexisting pluripotent stem cells [6,7]. The latter theory can be explained because melanocytes are derived from the neural crest and migrate along dorsolateral pathways during embryogenesis [10]. Thus, melanoma may be arrested in lymph nodes or benign nevus cells from the skin. More research is needed to further understand the pathogenesis of MUP.

Differential diagnoses for unilateral lymphadenopathy include nonspecific reactivity due to upper extremity infection or injury, lymphoma, sarcoma, melanomas, other metastatic malignancies, rheumatoid arthritis, systemic lupus erythematosus, Sjögren’s syndrome and dermatomyositis [11]. There are several methods to detect MUP. The first method is to perform a full physical exam including skin, ophthalmologic exam, otolaryngologic exam to identify a primary source [12]. If there are no known primary sources, mammograms and ultrasounds are warranted. Ultrasound has the highest sensitivity and specificity for regional lymph node basins [13]. PET-CT scans have a higher accuracy of detecting MUP, but has been suggested for guiding management [14,15]. Alongside imaging, immunohistochemistry markers such as HMB-45, MART-1/Melan-A, tyrosinase, and MITF demonstrate good specificity. S-100 marker is preferred because it has both good specificity and sensitivity. Ki67 remains the most useful adjunct in distinguishing benign from malignant melanocytic tumors [16].

Patients with MUP have similar disease course, prognostic factors, and treatment plans to patients with primary cutaneous melanoma [3]. In 2009, the American Joint Committee on Cancer classified MUP as stage III if presented with lymph node or subcutaneous involvement or stage IV with visceral involvement [4]. The preferred treatment plan for both stage III and stage IV MUP is aggressive surgical involvement and adjunct therapies including chemotherapy, immunotherapy, and radiotherapy [3,5]. Lee et al. showed that the median overall survival rate and five-year overall survival rate for stage IV in patients with MUP is significantly higher following aggressive treatment [5]. A non-surgical approach has a worse survival rate [17]. Routine follow-ups are instrumental as there is an 11% chance of recurrence even with lymph node dissection [18].

## Conclusions

Melanomas with an unknown primary are uncommon and the pathogenesis remains unclear. MUP must remain on the differential diagnosis in patients with unilateral lymphadenopathy without external breast changes. Proper physical examination, imaging, and histopathological review are needed to diagnose MUP. Patients with MUP must be aggressively treated with surgical resection and adjuvant therapy to improve survival rates.
